# Nimodipine, a calcium channel blocker, delays the spontaneous LH surge in women with regular menstrual cycles: a prospective pilot study

**DOI:** 10.1186/1477-7827-11-7

**Published:** 2013-02-07

**Authors:** Dan Nayot, Shany Klachook, Robert F Casper

**Affiliations:** 1 Department of Obstetrics & Gynaecology, Mount Sinai Hospital, University of Toronto, 92 College Street, Toronto, Canada; 2 Samuel Lunenfeld Research Institute, Mount Sinai Hospital, University of Toronto, Toronto, Canada

**Keywords:** Premature LH surge, In-vitro fertilization (IVF), GnRH agonist, GnRH antagonist, Calcium channel blocker, Nimodipine

## Abstract

**Background:**

Currently GnRH analogue injections are used to prevent premature LH surges in women undergoing assisted reproductive technology. This was a pilot study to determine the safety and effectiveness of nimodipine, an oral calcium channel blocker, to delay the mid-cycle spontaneous LH surge in women with regular menstrual cycles.

**Methods:**

Eight women with regular menstrual cycles self-monitored three consecutive cycles for the day of an LH surge by daily urine assay. The first and third cycles were observatory. In the second cycle, subjects took nimodipine 60 mg by mouth three times daily for four days, starting two days prior to the expected LH surge day based on cycle one.

**Results:**

The LH surge day in cycle 2 (nimodipine) was significantly delayed in comparison to both observatory cycle 1 (15.5+/−3.4 vs 14.0+/−2.8 days; p = 0.033) and cycle 3 (15.1+/−3.5 vs 13.1+/−2.4 days; p = 0.044). There was no difference in the LH surge day between the two observatory cycles (13.4+/−2.4 vs 13.1+/−2.4 days; p = 0.457). Three patients experienced a mild headache.

**Conclusions:**

There was a statistically significant delay in the spontaneous LH surge day in the treatment cycle in comparison to both observatory cycles. Nimopidine should be further investigated as an oral alternative to delay a spontaneous LH surge.

## Background

In reproductive-aged women regular menstrual cycles usually generate one mature ovarian follicle every month. In mid-cycle, a natural surge of luteinizing hormone (LH) and follicle stimulating hormone (FSH) is observed and predictably induces ovulation within approximately 36 hours. A critical step in the in-vitro fertilization (IVF) process is the properly timed aspiration of mature follicles prior to ovulation. Historically spontaneous ovulation was the major cause of treatment cancellation, occurring in up to 20% of cases, even in unstimulated IVF cycles [1]. Furthermore, a premature LH surge may lead to premature luteinization, which has detrimental effects on pregnancy rates [2,3]. Therefore, inhibiting spontaneous ovulation is critical for successful oocyte retrieval and is routinely integrated into IVF stimulation protocols. Current practice utilizes gonadotropin-releasing hormone (GnRH) agonists or antagonists that act centrally on the anterior pituitary gland to inhibit the release of LH and FSH, thus preventing spontaneous ovulation [4]. These medications are administered subcutaneously on a daily basis and are associated with prolonged treatment protocols, increased doses of gonadotropins for stimulation and added costs [5].

The intrinsic pulsatile secretion of GnRH has been directly associated with rhythmic changes in intracellular calcium concentration in GnRH neurons within the hypothalamus [6]. Recent evidence suggests that GnRH secretion can be markedly dampened using calcium channel blockers (CCB). It was noted that nifedipine, a CCB, inhibited the calcium dependent depolarization of clonal pituitary gonadotrophic cells in-vitro [7,8]. Furthermore direct injection of nifedipine into the preoptic area of the brain completely abolished the LH surge in proestrus rats [9]. However, due to its relatively hydrophilic nature, nifedipine has minimal ability to cross the blood–brain barrier to elicit an inhibitory effect on the GnRH neurons in clinical practice [10].

On the other hand, nimodipine (Nimotop; Bayer, Mississauga, ON) is a lipophilic CCB that is able to cross the blood–brain barrier [11]. Nimodipine is currently approved to reduce the severity of neurological deficits in patients who have had a recent subarachnoid hemorrhage (SAH) [12]. On a cellular level, nimodipine has been shown to inhibit the pulsatile activity of GnRH gene expression [13], an intrinsic property of GnRH neurons that is necessary for proper initiation of the LH surge. Specifically, nimodipine blocks L-type voltage gated calcium channels, which prevent the influx of extracellular calcium and subsequently the stimulation of GnRH release [8,14,15]. A recent animal study by our group demonstrated that oral administration of nimodipine to mice in the metaestrus phase resulted in a noticeable inhibition of ovulation in a dose related manner [16].

Due to its ability to cross the blood–brain barrier and suppress the calcium dependent pulsatile GnRH release, we proposed that nimodipine might inhibit the spontaneous mid-cycle LH surge and potentially serve as an inexpensive oral alternative to GnRH agonist or antagonist medications. The present study was a proof of principle pilot study to determine the safety and effectiveness of nimodipine to delay the mid-cycle spontaneous LH surge in reproductive-aged women with regular menstrual cycles.

## Methods

This was a prospective observational pilot study with Mount Sinai Hospital Research Ethics Board (MSHREB) approval (90-0175-A). Written informed consent was obtained from the participants of this study. Eight healthy reproductive-aged women with regular menstrual cycles were enrolled in the study.

Our inclusion criteria included women age ≥18 years old with regular menstrual cycles for a minimum of three consecutive months as defined by a cyclical pattern of menses ranging between 21 to 35 days or clinical evidence of monthly ovulation. We excluded women who were actively trying to conceive, currently pregnant, had a delivery within the last six months or actively breastfeeding. Any condition that could lead to non-ovulatory menstrual cycles, including the current use of hormonally based contraception of any form, was also an exclusion criterion.

Study subjects were recruited voluntarily from the community through advertisements at Mount Sinai Hospital, Toronto. The screening process for enrollment was conducted during scheduled appointments at the Toronto Center for Advanced Reproductive Technology (TCART) during the period of July 2010 to September 2010. An evaluation of the patient’s medical history was conducted to ensure all inclusion and exclusion criteria were met. All subjects were provided a complete package that included nimodipine capsules, LH urine test kits and a booklet to assist the patient in documentation and to facilitate accurate data retrieval (Table 1).

**Table 1:** Monitoring chart given to patients: mock chart completed

**Cycle**	**Day 10**	**Day 11**	**Day 12**	**Day 13**	**Day 14**	**Day 15**
**1**	LH -	LH -	LH -	**LH +**		
**2**		Nimodipine	Nimodipine	Nimodipine	Nimodipine	
				**Expected LH surge**		
	LH -	LH -	LH -	LH -	LH -	**LH +**
**3**	LH -	LH -	LH -	**LH +**		

Nimodipine was taken 2 days before the *expected LH* surge as determined by the 1st menstrual cycle and continued for a total of 4 days. In this mock patient, the LH surge was noted on Day 13 during the first and third cycle, but on Day 15 during the second (nimodipine) cycle.

Participants self-monitored three consecutive menstrual cycles with respect to menstrual onset and duration. The subjects also monitored the presence or absence of a natural LH surge using the LH urine test kit (First Response Easy Read Ovulation Test, Princeton, NJ) during all three cycles. The LH test was performed daily at the same time starting from the mid-follicular phase of the cycle until an initial positive test was detected.

The first and third cycles were completely observatory; self-monitoring booklet and LH urine test kit only. Each patient served as her own control, and an expected LH surge cycle day was determined based on the results of the first cycle. In the second cycle subjects took nimodipine 60 mg (two 30 mg capsules) orally three times daily for a total of four days (or until a positive LH surge was recorded), starting two days before the expected LH surge cycle day.

If a participant surged “prematurely” in their second cycle (prior to the initiation of nimodipine – two or more days prior to the expected LH surge day) then nimodipine was withheld and the cycle was used as an additional observatory cycle. In this scenario we used the average LH cycle day from the first two cycles as the new expected LH surge cycle day and the study resumed as per protocol. If a positive LH surge occurred in the four-day period of taking nimodipine (cycle 2), the medication was discontinued and the participant continued to self-monitor the cycle as planned.

The primary outcome measure was LH surge delay (cycle 2 positive urine LH day – cycle 1 or 3 positive urine LH day). Cycle length was calculated from the first day of menstruation to the subsequent first day of menstruation. Patient compliance with the medication and any side effects were documented using a standardized questionnaire. Data were analyzed using SPSS. Comparisons of LH surge day and cycle lengths between cycles were performed using paired t-tests.

Informed consent was obtained from all participants prior to the start of the study. Monetary compensation was provided only to cover travel expenses to TCART. Since this was a proof-of-principle pilot study on human subjects, we minimized drug exposure by using the lowest published effective dose and restricting the sample size under the guidance of the MSHREB.

## Results

**Figure 1:**
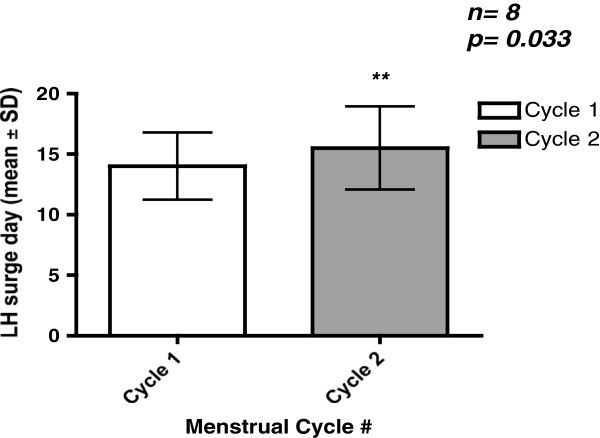
**LH surge of 8 female patients (mean ± SD).** The LH surge in the nimodipine cycle was significantly delayed in comparison to observatory cycle 1 (mean 15.5 ± 3.4 days vs 14.0 ± 2.8 days, p = 0.033). **Comparisons of LH surge day between cycles were performed using paired t-tests.

**Figure 2:**
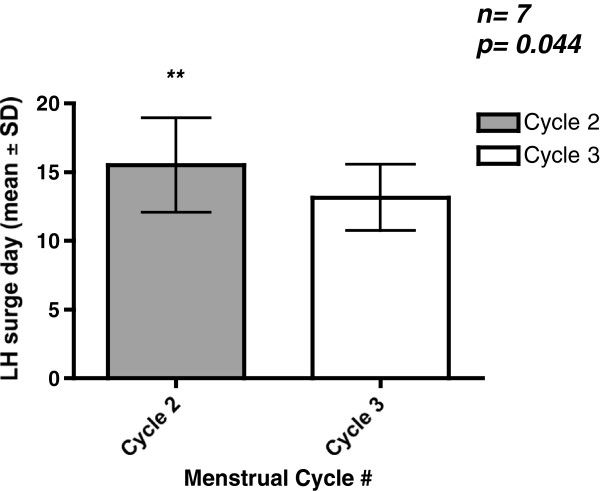
**LH surge of 7 female patients (mean ± SD).** The LH surge was significantly delayed in the nimodipine cycle 2 in comparison to observatory cycle 3 (mean 15.1 ± 3.5 days vs 13.1 ± 2.4 days, p = 0.044). Among the eight patients, one patient had an anovulatory 3rd cycle and did not have a positive LH surge. **Comparisons of LH surge day between cycles were performed using paired t-tests.

There were a total of eight patients who fulfilled study criteria with an average age of 35.8 +/− 7.5 years old. There were 23 monitored cycles with a documented LH surge as one patient had a presumed anovulatory 3rd cycle (absence of an LH surge). Five out of eight patients had their spontaneous LH surge delayed by at least one day during the nimodipine cycle (range 0–4 days) in comparison to the initial observatory cycle (Table 2). The LH surge was significantly delayed in the nimodipine cycle relative to both observatory cycle 1 (N = 8, 15.5+/− 3.4 days vs 14.0+/− 2.8 days, p = 0.033; Figure 1) and cycle 3 (N = 7, 15.1+/− 3.5 days vs 13.1+/− 2.4 days p = 0.044; Figure 2). There was no difference in the LH surge day between cycle 1 and cycle 3 (N = 7; 13.4+/− 2.4 days vs N = 7; 13.1+/− 2.4 days; p = 0.457).

**Table 2:** LH surge day, by cycle

**Patient ID**	**Cycle 1: LH surge day**	**Cycle 2: (Nimodipine) LH surge day**	**Cycle 3: LH surge day**	**LH surge delay cycle 2 – cycle 1 [days]**
1	11	11	12	0
2	13	14	11	1
3	15	15	15	0
4	13	16	13	3
5	18	18	N/A	0
6	17	20	17	3
7	15	19	14	4
8	10	11	10	1

There was no statistical difference in the mean cycle lengths between the interventional cycle (cycle 2: mean 28.4+/− 2.8 days) and either of the observational cycle 1 (mean 28.1+/− 2.7 days; p = 0.598) or cycle 3 (mean 27.4+/− 2 days; p = 0.259). No menstrual cycle irregularities were reported in the nimodipine cycle. In the third cycle one patient had an anovulatory cycle and one patient reported two days of spotting after their LH surge, while the rest of the participants reported regular cycles throughout the study.

There was complete medication compliance among study participants. Three out of eight subjects experienced headaches that were temporally associated with nimodipine administration. Headaches began within 1–2 hours of taking the drug (n = 2) or that same evening (n = 1). The duration of the headaches was on average 2.2 days (range 1–4 days) and the pain level was rated an average of 5.3 on a scale of 0–10 (range 4–7). Over the counter medication was effective in alleviating headaches in all subjects and their symptoms subsided upon discontinuation of nimodipine. One patient reported brief shortness of breath and tachycardia temporally associated with pill intake. Symptoms were more intense in the morning and were alleviated slightly by reducing caffeine intake. Another patient reported fatigue and drowsiness that she believed to be a result of nimodipine ingestion, which was more severe at night. Both subjects reported that their symptoms abated upon discontinuation of medication. However, all participants classified nimodipine as a medication with tolerable side effects.

## Discussion

Our results show that there was a significant delay in the spontaneous LH surge in the cycle in which patients took nimodipine in comparison to both observatory cycles, with no effect on overall cycle length. Furthermore nimopidine was generally tolerable, with only mild and self-resolving side effects.

Due to MSHREB restrictions our study design focused on assessing the safety and effectiveness at the lowest therapeutic dose and duration in the minimum number of required participants. While patients took nimodipine for a period of four days, only two days of treatment occurred beyond the expected LH surge day based on the first cycle (Table 1). Therefore complete efficacy in delaying the LH surge represented a maximum two-day delay in the treatment cycle. In total, five out of eight patients had an intended delay of a spontaneous LH surge by at least one day during their nimodipine cycle. For the remainder of the patients the LH surge day was the same as the first cycle. None of the patients had an LH surge in the two day window prior to the expected LH surge day (Table 2).

Patients undergoing intra-uterine insemination (IUI) cycles, with or without ovarian stimulation, don’t routinely utilize any medical suppression of the natural LH surge. Not surprisingly, these cycles have a high prevalence of premature LH surge, commonly defined as a natural LH surge occurring before the leading ovarian follicle reaches 18 mm in diameter. In a recent study of 87 monitored IUI cycles patients were administered clomiphene citrate (CC) (60%), letrozole (16%) or no ovarian stimulation (24%), and 28.7% had a premature LH surge, with no significant difference among the three groups [17]. In a similar study, patients were randomized to CC or recombinant follicle-stimulating hormone (rFSH) coupled with IUI. Among 153 monitored cycles 36% demonstrated a premature LH surge [18].

Given the prevalence of premature LH surge with ovarian stimulation, medical suppression of the physiological LH surge is routinely used in IVF cycles, which results in lower cancellation rates and improvement in routine organization. In current practice the LH surge is inhibited by GnRH agonists or antagonists, both of which ultimately suppress pituitary gonadotropin secretion [4]. Both these treatment options increase the need for multiple injections resulting in increased patient discomfort, inconvenience and overall costs [5]. Hence, an inexpensive oral medication, such as nimodipine, would be welcomed as a more convenient and cost effective approach if shown to be efficacious in preventing premature LH surges in ART protocols. Even in IUI cycles, where ovulation suppression is not used, there may be a pregnancy benefit to delaying the LH surge. A recent publication has demonstrated that the optimal follicle size on ultrasound to achieve pregnancy in clomiphene citrate or letrozole cycles is 2.5 cm rather than 1.8 cm [19]. Nimodipine is a relatively lipophillic calcium channel blocker that crosses the blood brain barrier to exert its effects intracerebrally [11]. This property makes nimodipine an ideal candidate for an oral medication to affect GnRH neurons. There is strong evidence that nimodipine can suppress GnRH release on a cellular level [8,14,15] and prevent ovulation in mice [16].

Numerous other hormones [20-23], peptides [24,25] and endotoxins [26] have been explored as candidates to suppress a premature LH surge, but studies have been restricted to animal studies and clinical evidence is lacking.

Several reproductive-associated hormones, such as progesterone and mifepristone (anti-progesterone) have been investigated in human subjects. One clinical trial examined the effect of a five-day mid-cyle course of ethinyl estradiol and norethindrone on 10 volunteers with regular ovulatory cycles. None of the patients had an LH surge or evidence of ovulation, while ultrasound findings suggested appropriate follicular growth and endometrial thickening [27]. In another small study of nine patients serving as their own controls, subjects were stimulated with rFSH in the initial cycle and with FSH and mifepristone in the subsequent cycle. An endogenous LH surge was noted in 6/9 patients in the first cycle, but none in the second. The major drawback was mifepristone’s adverse effect on folliculogenesis and decreased estradiol and progesterone levels in the luteal phase [28]. A more recent pilot study of 15 healthy oocyte donors undergoing ovarian stimulation for IVF investigated the effectiveness of daily mifepristone in comparison to GnRH agonist to prevent premature LH surge. Although no statistical analysis was published, it was noted that none of the ten subjects treated with 40 mg mifepristone daily had a premature surge while one of the five GnRH agonist patients had premature luteinization. However, endometrial biopsies in these patients demonstrated that mifepristone had a negative impact on endometrial receptivity at the gene level, even with progesterone supplementation [29]. The clinical utility of progesterone and mifepristone supplementation during an ovarian stimulation cycle, with the aim of inhibiting an LH surge, must be questioned as it may mimic premature luteinzation or adversely affect implantation [2,3]. There is currently no effective alternative to GnRH analogues to inhibit the spontaneous LH surge in women undergoing ovarian stimulation.

It is imperative to keep in context the limitations of our single-arm pilot study composed of a small sample size of unblinded healthy participants. On the other hand, there does appear to be a significant temporal association between nimodipine and LH surge delay as patients served as their own controls, pre and post-intervention. Furthermore the rationale to use nimodipine to inhibit an LH surge is solidly based on both in vitro and in vivo animal research. Unlike other medications investigated in clinic settings, nimodipine is not a hormone and thus may have a more isolated effect on the LH surge, although this has yet to be proven. Furthermore it is an inexpensive oral medication with a high safety profile that could potentially be used to prevent a spontaneous LH surge.

In our study subjects received 60 mg only every eight hours for a total of four days and there were no reported cases of severe side effects. While three out of eight patients experienced a headache that was associated with nimodipine intake, conservative measures were effective in reversing symptoms, which subsided upon discontinuation of nimodipine. Without a placebo comparison arm it remains difficult to ascertain whether this is the direct effect of the medication, although it appeared to be temporally associated. In two double-blinded randomized controlled trials comparing nimodipine to placebo there was no difference in side effects [30] and even an increased adverse event rate in the placebo arm (RR = 1.29; CI 1.03-1.61) [31]. Interestingly, of the three patients that experienced a headache, one had an LH surge delay of three days and another of four days.

Study participants all had a history of regular menstrual cycles, but there remains a natural intra-patient variability of LH surge day. We restricted our study design to investigate a two day delay in order to minimize drug exposure in this pilot study. It is noteworthy that nimodipine was initiated two days prior to the anticipated LH surge and no patients in the medication cycle had an LH surge in the two day window prior to the expected date. As well there was no statistical difference in the LH surge day between the two control cycles further supporting the stability of the LH surge day in our subjects.

One drawback to our study design is that if patients had an LH surge prior to initiating nimodipine (expected LH surge – 2 days), the interventional cycle was suspended and the data was used as part of a second observatory cycle, although this only occurred in one patient.

The primary outcome of this study was the LH surge day. A delayed LH surge, in essence a prolonged follicular phase, is expected accompany a longer menstrual cycle. Interestingly our results demonstrated no difference in cycle length in the nimodpine cycle. We have no explanation for this observation, but perhaps with an intentional longer LH surge delay we will be able to identify a corresponding difference in cycle length.

## Conclusions

This proof of principle study served to confirm the safety and effectiveness of nimodipine in delaying the spontaneous LH surge in women with regular menstrual cycles. This study has now led to a RCT that is currently underway to investigate the clinical use of nimodipine as an alternative to delay the premature LH surge in women with subfertility undergoing ovarian stimulation cycles and IUI.

## Abbreviations

LH: Luteinizing hormone;FSH: Follicle stimulating hormone;IVF: In-vitro fertilization;GnRH: Gonadotropin-releasing hormone;CCB: Calcium channel blocker;SAH: Subarachnoid hemorrhage;MSHREB: Mount sinai hospital research ethics board;TCART: Toronto center for advanced reproductive technology;IUI: Intra-uterine insemination;CC: Clomiphene citrate;rFSH: Recombinant follicle-stimulating hormone;hMG: Human menopausal gonadotropins;RCT: Randomized controlled trial

## Competing interests

The authors declare that they have no competing interests.

## Authors’ contributions

DN participated in the design of the study, grant and research ethics application, and helped to draft the manuscript. SK coordinated the study, performed data collection and helped draft the manuscript. RFC was the principal investigator, conceived the study and helped to draft the manuscript. All authors read and approved the final manuscript.
